# Prototype Positive Control Wells for Malaria Rapid Diagnostic Tests: Prospective Evaluation of Implementation Among Health Workers in Lao People's Democratic Republic and Uganda

**DOI:** 10.4269/ajtmh.16-0498

**Published:** 2017-02-08

**Authors:** David Bell, John Baptist Bwanika, Jane Cunningham, Michelle Gatton, Iveth J. González, Heidi Hopkins, Simon Peter S. Kibira, Daniel J. Kyabayinze, Mayfong Mayxay, Bbaale Ndawula, Paul N. Newton, Koukeo Phommasone, Elizabeth Streat, René Umlauf, Malaria RDT Positive Control Well Field Study Group

**Affiliations:** 1 The Global Good Fund/Intellectual Ventures Lab, Bellevue, Washington.; 2 Malaria Consortium, Kampala, Uganda.; 3 World Health Organization Global Malaria Programme, Geneva, Switzerland.; 4 Queensland University of Technology (QUT), Brisbane, Australia.; 5 Foundation for Innovative New Diagnostics (FIND), Geneva, Switzerland.; 6 Foundation for Innovative New Diagnostics (FIND), Kampala, Uganda.; 7 Makerere University School of Public Health, Kampala, Uganda.; 8 Lao-Oxford-Mahosot Hospital-Wellcome Trust Research Unit (LOMWRU), Microbiology Laboratory, Mahosot Hospital, Vientiane, Lao People's Democratic Republic.; 9 Faculty of Postgraduate Studies, University of Health Sciences, Vientiane, Lao People's Democratic Republic.; 10 Centre for Tropical Medicine and Global Health, Churchill Hospital, University of Oxford, Oxford, United Kingdom.; 11 University of Bayreuth, Bayreuth, Germany.

## Abstract

Rapid diagnostic tests (RDTs) are widely used for malaria diagnosis, but lack of quality control at point of care restricts trust in test results. Prototype positive control wells (PCW) containing recombinant malaria antigens have been developed to identify poor-quality RDT lots. This study assessed community and facility health workers' (HW) ability to use PCWs to detect degraded RDTs, the impact of PCW availability on RDT use and prescribing, and preferred strategies for implementation in Lao People's Democratic Republic (Laos) and Uganda. A total of 557 HWs participated in Laos (267) and Uganda (290). After training, most (88% to ≥ 99%) participants correctly performed the six key individual PCW steps; performance was generally maintained during the 6-month study period. Nearly all (97%) reported a correct action based on PCW use at routine work sites. In Uganda, where data for 127,775 individual patients were available, PCW introduction in health facilities was followed by a decrease in antimalarial prescribing for RDT-negative patients ≥ 5 years of age (4.7–1.9%); among community-based HWs, the decrease was 12.2% (*P* < 0.05) for all patients. Qualitative data revealed PCWs as a way to confirm RDT quality and restore confidence in RDT results. HWs in malaria-endemic areas are able to use prototype PCWs for quality control of malaria RDTs. PCW availability can improve HWs' confidence in RDT results, and benefit malaria diagnostic programs. Lessons learned from this study may be valuable for introduction of other point-of-care diagnostic and quality-control tools. Future work should evaluate longer term impacts of PCWs on patient management.

## Introduction

Rapid diagnostic tests (RDTs) are now widely used for malaria diagnosis, consistent with World Health Organization (WHO) recommendations for areas where good-quality malaria microscopy is not available, including peripheral health facilities and community-based fever management programs.^1^,^2^ The need for stable, high-performing RDTs, especially under transport and storage conditions typical in malaria-endemic regions, has received considerable attention.^3^–^5^ RDT lot-to-lot variation and susceptibility to deterioration upon exposure to high temperatures and humidity in supply chains have been documented.^6^,^7^ In addition, some reports attribute health workers' poor adherence to RDT results at least in part to lack of confidence in test results.^8^,^9^ To maintain confidence in RDTs and optimize their utility, the tests must demonstrate consistently reliable results. However, RDT quality control, after field deployment, is currently difficult to implement in routine health-care contexts.^10^–^12^

A global program supports quality assurance activities for malaria RDTs through independent laboratory-based assessment of commercially available products manufactured under ISO13485, lot verification of procured RDTs, and provision of training materials.^13^ Positive control wells (PCWs) have been proposed as point-of-care quality-control tools, as a third component of a tiered quality assurance program.^14^–^17^ Prototype PCWs have been developed as single-use plastic wells containing small amounts of recombinant malaria parasite antigens targeted by commercially available RDTs. When reconstituted with water and applied to a good-quality RDT, the antigen solution produces a positive reaction on the RDT. PCWs can therefore be used to test stocks of RDTs stored and used at health facilities, to ensure their validity. PCWs may also be used to monitor RDT quality along the supply chain.

The study described here is part of a step-wise approach to collect evidence to guide rational implementation strategies for PCWs. The present study was designed to determine whether health workers in malaria-endemic settings can use PCWs correctly to detect RDTs with inadequate sensitivity after a half-day training, to assess the impact of PCW availability on RDT use, and to gather information on health workers' perceptions of PCWs and preferred strategies for routine use in public health-care sectors.

## Methods

### Ethics and protocol.

All participating health workers provided written informed consent. Before participant recruitment, the study protocol was approved by the National Ethics Committee for Health Research, Lao People's Democratic Republic (Laos) (NECHR 009/2012); Oxford Tropical Research Ethics Committee of the University of Oxford, United Kingdom (1000-13); Vector Control Division Ethical Committee of the Uganda Ministry of Health (VCD-IRC/038); Uganda National Council for Science and Technology (HS 1271); and Research Ethics Review Committee of the World Health Organization (protocol ID RPC545).

### Study sites and setting.

The study was conducted from March to October 2013 in Salavan Province, southern Laos, and in Kiboga District, west-central Uganda. Study area selection criteria were malaria RDTs meeting WHO procurement criteria^18^ already in routine use in clinical care according to plans/programs approved by the national malaria control authorities, representative sites in Africa and Asia, and local collaborators experienced in the conduct of operational research on malaria diagnosis.

Malaria transmission in Salavan Province is highly seasonal, typically beginning around June and peaking during and after the annual rainy season (Lao Center of Malariology, Parasitology and Entomology [CMPE], unpublished data). Malaria transmission in Kiboga District is moderately high year round (proportion of malaria blood slides positive in fever cases was 40–60% [Uganda Ministry of Health, unpublished data]). Before the study started, 65–95% of fever patients were RDT negative in southern Laos, depending on season, whereas 40–60% of fever cases were RDT negative in midwestern Uganda. The study was conducted at government-sponsored health facilities and at community or village health volunteers' work stations where RDTs are used in routine patient care.

In addition, to assess the impact of PCW availability on RDT use, in each country, routine clinical data from a neighboring “control” area with similar climate, malaria epidemiology, health-care infrastructure, and RDT access but without PCWs (Sekong Province in Laos; Kyankwanzi District in Uganda) were obtained as aggregate summaries from the Ministry of Health (Laos) or from individual health facility and community worker logbooks (Uganda).

RDTs used in this study were provided through routine procurement and distribution mechanisms in each country. In Laos, RDTs are provided to government health facilities and village health volunteers by CMPE, Lao Ministry of Health. The RDTs in use at the time of this study were SD Bioline Malaria Antigen Pf/Pv (Standard Diagnostics, Youngin-si, Gyeonggi-do, Republic of Korea) (catalogue no. 05FK80, lot 082171). In Uganda, RDTs were provided in the study area by a project led by the Malaria Consortium. The RDTs in use at the time of this study were SD Bioline Malaria Antigen Pf (catalogue no. 05FK50, lot 082140). Before study activities began, RDTs from each study area passed lot testing at WHO and Foundation for Innovative New Diagnostics (WHO-FIND)–recognized lot testing laboratories.^19^

### Study population.

Basic health care in the study areas is provided by staff of health facilities (“clinic staff” in this report), typically nursing and clinical staff with < 2–3 years of formal training; and by village or community health volunteers (“community workers”), literate or semiliterate volunteers with a few weeks' training who work at or near their own home. The term “health worker” is used here to include both clinic staff and community workers. Within the study areas, health workers were invited to participate if their work place met these selection criteria: established use of RDTs in routine clinical work as the only parasite-based malaria diagnostic method (i.e., no microscopy capacity); at least five patients seen per month; and availability of records or logbook with data on RDT use, patient diagnoses, and treatments.

### Sample size.

A sample size of approximately 300 health workers in each of the two study areas was targeted to participate and receive PCWs. The goal was to include a representative sample of health workers who use malaria RDTs in routine practice, with recruitment of approximately 225 community workers in each country and the remainder being clinic staff. The target sample size represented approximately 3–5% of the community workers using RDTs in each country.

### Prototype PCW.

**Figure 1.**
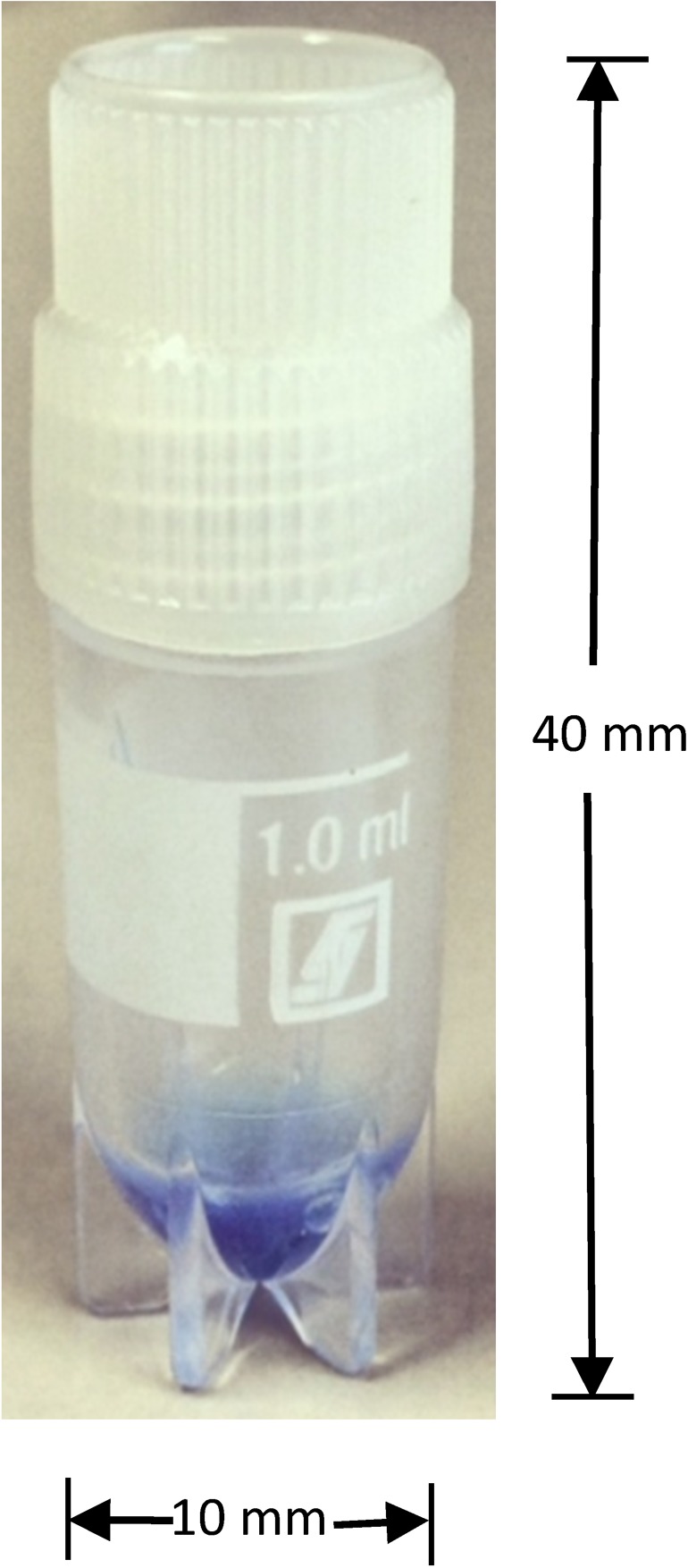
Prototype positive control well (PCW) for malaria rapid diagnostic tests.

The prototype PCW used was developed by FIND, Geneva, Switzerland, in partnership with ReaMetrix Inc., Bangalore, India. The product specifications of the PCW were single-use, disposable, free-standing individual tube containing dried recombinant antigens, synthetic variants of the malaria parasite antigens targeted by commercially available RDTs, that is, histidine-rich protein 2, parasite lactate dehydrogenase, and aldolase (Figure 1

). The PCW contained a sufficient concentration of each antigen to produce a test line on a well-performing RDT, whereas failing to produce a line on an RDT that has deteriorated to a point unreliable for detection of clinically significant parasitemia (∼200 parasites/μL).^20^

To perform a PCW, antigens were reconstituted by adding 100 μL of water (e.g., handwashing water) to the tube and stirring for 2 minutes using a squeezable pipette packaged with the PCW (see pictorial guide, Supplemental online material). The desired amount of PCW solution, that is, 5 μL, was placed in the RDT sample well using the transfer device packaged with the RDT, and RDT buffer was added. The wicking speed along the nitrocellulose strip was similar to lysed blood and the test results were read according to RDT instructions. PCWs were stored in their original packaging at ambient temperature at the local offices/laboratories of collaborating research organizations in each country before study activities began, and at health worker work sites and homes during the study.

### PCW training and study initiation.

All training and data collection tools are in the Supplemental online material. An initial 1-week pilot assessment preceded the study, during which a pictorial guide (job aid) for PCW interpretation was developed for use in both Uganda and Laos. PCWs were introduced to participating health workers with a standardized half-day training package presented by members of the study team, who were individuals with laboratory and/or clinical background and with prior experience in clinical malaria research and/or program implementation. Trainings were typically held for groups of 12–20 health workers at a central point in each subregion within the study areas. No training in RDT use or fever case management was provided as part of this study.

After the training and initial assessment, PCWs were given to each participating health worker, along with forms for recording PCW use. Health workers were not given specific guidance on when or how frequently to use PCWs; they were told that they could use a PCW whenever they felt it was appropriate. Health workers were provided with phone numbers of study staff and encouraged to call with questions, especially if a negative or invalid RDT result was obtained with a PCW during routine use. Study staff returned calls so that there was no cost to health workers.

### Assessment of health workers' performance, interpretation, and use of PCWs.

**Figure 2.**
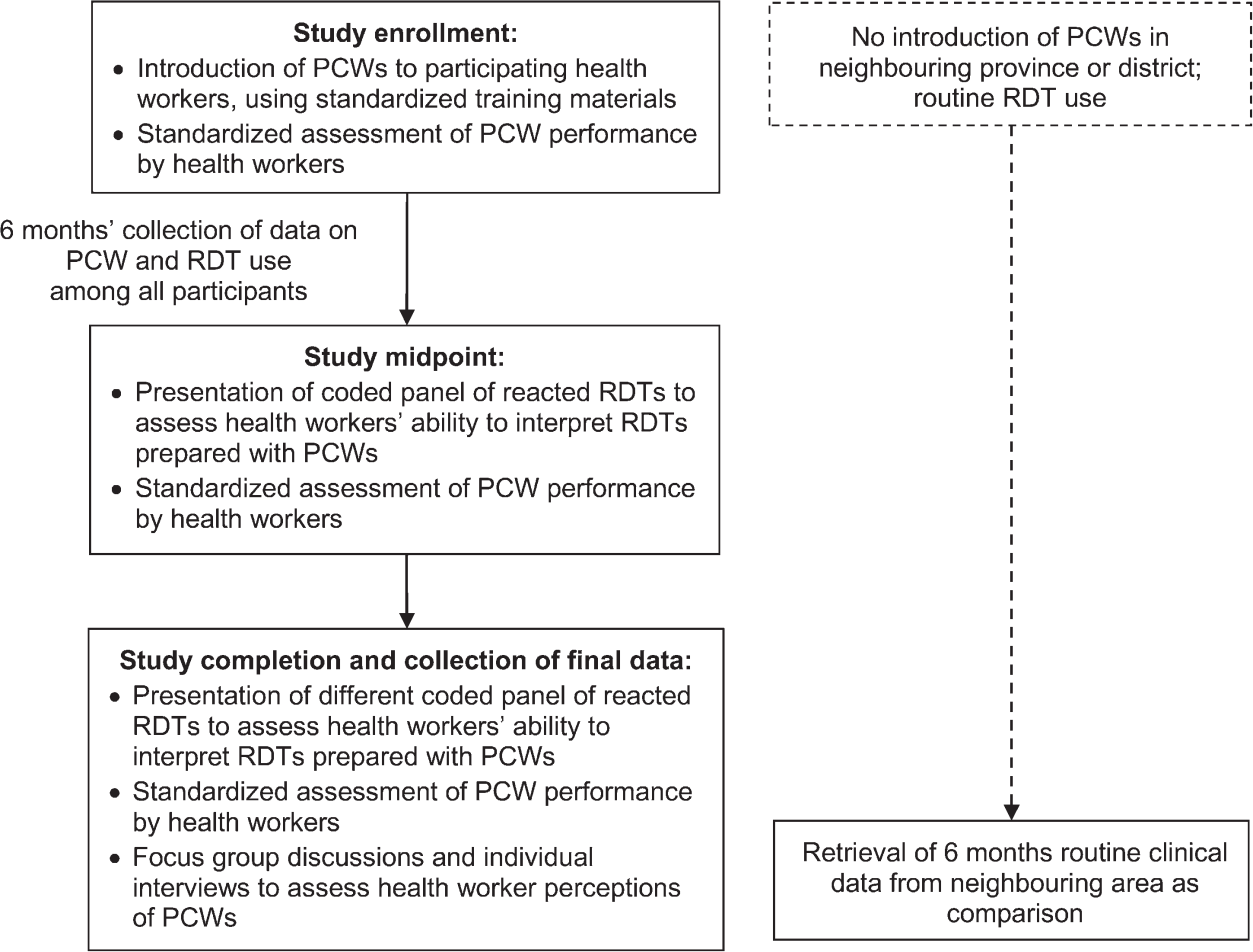
Study flow diagram. Study activities and data collection: In each of the two study areas, one province in Lao People's Democratic Republic and one district in Uganda, a target sample of approximately 300 health workers was recruited to participate in the study. Participants were trained in positive control well (PCW) use, and supplies of PCWs were left at each work site. Data collection continued for 6 months after the introduction of PCWs. Routine clinical and rapid diagnostic test (RDT) use data from a neighboring area in each country, without PCWs, were retrieved as a comparison.

After the initial training, health workers' ability to correctly use PCWs was assessed using three approaches at three time points: immediately after training, at the study midpoint about 3 months later, and at the end of the study 6 months after training (Figure 2

). First, the study team used a standardized checklist to observe and score individual participants on PCW performance and result interpretation. Health workers had free access to the PCW job aid, and any mistakes or questions were addressed after the health worker had completed all steps, to avoid biasing the assessment. Second, at the study midpoint and endpoint, each health worker was individually presented with panels of reacted RDTs and asked to propose the correct actions if they obtained these results with a PCW. Third, the forms completed by health workers during their routine work over the study period were retrieved to determine: 1) frequency of use of PCWs, 2) results of RDTs tested with PCWs, 3) interpretation of results, and 4) any actions taken.

### Assessment of impact of PCW availability on RDT use.

In Laos, aggregated data on RDT use, results, and treatments prescribed were obtained through CMPE from Salavan Province, and from neighboring Sekong Province (control). Logbook data, handwritten by health workers, were transferred to the central level for computerized data entry. CMPE provided summary data from the 6-month study period and from the 3 months preceding it.

In Uganda, patient-level data were obtained from participating health facilities and community workers in Kiboga District and from neighboring Kyankwanzi District (control). Logbook data from the study period and the preceding 3 months, handwritten by health workers, were transferred to district level for routine reporting and filing and entered into a computerized database. Data retrieved included patient age, gender, RDT result (if done), diagnosis made, and treatment prescribed.

### Assessment of health workers' perceptions of PCWs.

At the end of the 6-month study period, focus group discussions (FGDs) and individual semistructured interviews were held to gather qualitative information on health workers' experiences with and perceptions of PCWs. Health workers were purposively selected for participation to achieve representation from clinic staff and community workers, geographical subregions within the study areas, demographic features, and a range of observed abilities to correctly use PCWs. Discussions followed topic guides developed for this purpose (Supplemental online material), and were conducted in local languages.

### Data management and statistical analysis.

Quantitative data were double entered using Microsoft Office Excel 2007 (Microsoft, Redmond, WA) in Laos and EpiData (EpiData Association, Odense, Denmark) in Uganda. Stata version 9 (StataCorp, College Station, TX), and SPSS version 23 (IBM Corporation, New York City, NY) were used for quantitative data analysis. Training outcomes were presented as proportions and frequencies. Comparisons between groups were made using Pearson's χ^2^ or Fisher's exact test, whereas changes in performance between assessments were assessed using either McNemar or McNemar–Bowker test. Binary logistic regression was used to assess the association between age and amount of time the participant had been using RDTs on correctly preparing individual PCW steps and interpreting RDT results. Poisson regression was used to assess the association between age, facility, and PCW use on the proportions of patients tested by RDT, positive by RDT, and RDT-positive patients treated with an antimalarial. Estimated marginal means, along with the 95% confidence intervals (CIs), were calculated by the statistical software and used to illustrate the proportion of patients tested by RDT, positive by RDT, and RDT-positive patients treated with an antimalarial, after adjusting for significant confounders.

For qualitative data, FGD and interview audio files were transcribed into text files and translated into English. Analysis was performed with NVIVO QDA Mac Beta 2014 software (QSR International, Melbourne, Australia) to group key findings into themes and subthemes using content analysis.^21^ Themes that emerged from the data were categorized around local concepts of quality control and quality assurance.

## Results

A total of 267 health workers were enrolled in the study in Laos, and 290 in Uganda (Table 1). The majority were community workers (72% in Laos, 83% in Uganda), with the remainder being facility-based clinical or laboratory staff.

### Assessment of health workers' performance, interpretation, and use of PCWs.

#### Observed performance of PCWs.

Table 2 summarizes health workers' performance of PCWs as observed by study staff using the standardized checklist. The majority (88% to ≥ 99%) of participants correctly performed the six key individual PCW steps. Steps that appeared challenging for some participants included filling the PCW dropper with the correct amount of water, mixing the PCW solution for 120 seconds by counting or using a timer, and transferring a single drop of PCW solution to the correct RDT well. Observers' notes (not shown) indicated that apparently poor eyesight, and in some cases, limited finger dexterity, contributed to some health workers' difficulties with the dropper; errors included filling the dropper with water to either above or below the indicator mark. Errors in mixing included stirring both for too short a time and for too long. Common errors in transferring solution to the RDT included struggling or failing to collect a drop of solution from the PCW tube with the RDT transfer device, or adding more than one drop of solution; in the latter case, some participants mentioned that this was intentional, as they had noticed that adding more solution gave a stronger RDT test line.

When all six key steps in the PCW preparation procedure were considered together, the proportion of participants completing all steps correctly ranged from 62% to 93%. When errors were made, the majority (67–79%) of participants made only one error in the six steps, but the incorrect step varied between participants. In both study areas, the lowest composite performance occurred at the study midpoint (Table 2).

The proportion of health workers who correctly performed all six key PCW steps was not influenced by whether the participant was a community worker or clinic staff (*P* > 0.08), nor by how long the participant had been using RDTs in routine patient care (*P* > 0.15). Overall, the proportion of Ugandan participants who correctly performed all key steps was significantly lower than the Lao participants (*P* < 0.05), with the difference increasing over time. (Anecdotally, study staff noticed that the Uganda study team tended to be stricter in scoring than the Lao study team, so it may not be appropriate to compare the two sites on this outcome). Increasing health worker age was associated with an increase in the odds of incorrectly filling the PCW dropper in Ugandan participants at all assessments, with odds ratios (ORs) varying between 1.03 (95% CI = 1.00–1.07) at the initial assessment and 1.05 (95% I = 1.01–1.09) at the final assessment. In Laos, age was only significant at the initial assessment where the odds of incorrectly performing this step increased 1.07 (95% CI = 1.02–1.13)-fold for each year increase in participant age. There was no evidence of an age effect in this step during the other assessments (*P* > 0.8) in Laos.

Health workers had free access to the job aid while performing the PCW under observation (Table 2). In Laos, there was no difference in the frequency of referral to the job aid between community workers and facility-based staff (*P* > 0.1); however, in Uganda, a higher proportion of community workers referred to the job aid compared with facility-based staff, particularly in the midpoint and study end assessments (*P* < 0.01). In both countries at all assessments, there was no significant association between referral to the job aid during the assessment and correctly performing all six key steps (*P* > 0.2).

At all three assessment points in both countries, ≥ 97% of participants for whom data was recorded correctly read the result of the RDT they prepared with a PCW, and ≥ 98% gave a rational explanation for the result obtained. Errors in reading included confusion between positive and negative results or terminology, and failure to read faint lines as positive. Errors in explaining the result included both reporting that a positive result indicated a poor-quality RDT stock, and reporting that a negative or invalid result indicated a good-quality RDT stock.

#### Interpretation of panels of reacted RDTs.

Table 3 shows health workers' interpretation of reacted RDTs. At the study midpoint, the proportion of health workers who gave correct responses for all five RDTs was similar in both Laos and Uganda (89%, *P* > 0.9). At the study end, the proportion declined to 80% in Laos, whereas in Uganda, it increased to 93% (*P* < 0.001). Within each country, the change between the midpoint and study end was not significant (*P* > 0.09). In Laos, 75.3% of participants responded correctly for all five RDTs on both occasions, 2.5% made errors on both occasions, 14.6% were correct at the midpoint but made at least one error at study end, and 7.6% made errors at the midpoint but not at study end. In Uganda, these values were 83.4%, 1.9%, 5.7%, and 9.1%, respectively.

Errors were made in responses to positive, negative, and invalid tests. However, most participants recognized invalid tests as indicating the need for corrective action (97–99% across both sites and evaluation points). A faint positive RDT line presented at the study end presented a particular challenge (89% in Laos and 95% in Uganda responded correctly).

In Laos, there was no difference between the proportion of community workers and clinic staff who correctly interpreted all five RDTs (*P* > 0.08). In contrast, in Uganda at the study midpoint, more community workers correctly interpreted all five RDTs correctly (91%) than clinic staff (78%; *P* = 0.022). In both countries, neither age nor time spent using RDTs was associated with correct interpretation of RDTs (for Laos, *P* > 0.2; for Uganda, *P* > 0.3). There was a positive association between participants' ability to correctly interpret all five RDTs and to correctly perform the six key steps in PCW preparation in both countries (analysis not shown).

#### Use of PCWs during routine clinical work.

Records on PCW use during routine work over the study period were available from 221 (83% of total enrolled) to 275 (95%) participants in Laos and Uganda, respectively (Table 4). The number of PCWs used was not associated with the length of time a health worker had been using RDTs (Spearman's rank correlation, *P* > 0.2).

In Laos, the most common reason given for performing a PCW (481, 64%) was that the health worker had received a new stock of RDTs. Performing a PCW because of concerns about RDT results obtained with patients was not associated in Laos with type of health worker (*P* = 0.40), but it was somewhat more likely among those who had been using RDTs for a longer time (*P* = 0.06, OR = 1.01 [95% CI = 1.00–1.03]). In Uganda, the primary reason given (1,049, 64%) was to check the quality of existing RDT stocks. In Uganda, performing a PCW because of concerns about patients' RDT results was associated with type of health worker (*P* < 0.001, 16% in clinic staff versus 5% in community workers); here this reason was somewhat less likely among health workers who had been using RDTs for a longer time (*P* < 0.001, OR = 0.973 [95% CI = 0.958–0.987]). Some Ugandan participants wrote in other reasons for performing a PCW at their work site, including practicing or “reminding myself” of the PCW procedure, testing RDTs that were near or past their expiry date, or repeating a PCW test after an initial negative or invalid result.

Most records reported a correct action following use of a PCW at the routine work site, based on the RDT result obtained. In Laos, 97% of reported actions were correct. In Uganda, some participants wrote their action on the record form rather than ticking one of the choices on the form. In these cases, it was necessary to interpret the meaning from incomplete phrases and then categorize actions as “probably correct” or “probably not correct”; thus, 94% of actions were categorized as correct, and 99% as “correct or probably correct.” In Laos, clinic staff were slightly more likely than community workers to record a corrective action (99% versus 96%, *P* = 0.013), whereas in Uganda, there was no difference (*P* > 0.9). There was no association between reporting a correct or probably correct action and the length of time a health worker had been using RDTs in either country (*P* > 0.5). Reported actions were more often correct if the RDT result obtained with a PCW was positive than if the result was negative or invalid.

### Impact of PCW availability on RDT use.

In Laos, when aggregated data from clinic staff were compared between the PCW and control provinces, there were significant differences in the proportion of patients receiving an RDT in Salavan versus Sekong (*P* < 0.001), and also between patient age groups within each province (*P* < 0.001; Table 5). However, there was no difference in the rate of RDT use between the pre-PCW period (December 2012–March 2013) and the PCW period (April–November 2013) in either province (*P* > 0.6). In Salavan, the relative risk of receiving antimalarial treatment in a health facility, adjusted for the number of positive RDTs, was 1.04 (95% CI = 1.03–1.06) times higher after PCW introduction (April–November) compared with before PCW introduction (December–March) (*P* < 0.001; Table 5). No change in treatment rates by clinic staff were detected in Sekong between these same periods (*P* = 0.14). Data for community workers in Salavan and Sekong list only patients who were tested with RDTs (i.e., the proportion tested was 100%) and report that 100% of RDT-positive patients were treated with artemisinin-based combination therapy; no further analysis is possible.

In Uganda, individual patient data were compared between the PCW and control districts, stratified for management by clinic staff and community workers. Clinic staff performed a total of 60,144 RDTs for 87,893 patients. The proportion of patients tested was significantly higher in the control district (Kyankwanzi) than in Kiboga, and was also significantly higher in the pre-PCW period in both districts (Table 6). In the control district, the odds of receiving antimalarial treatment of positive RDT results increased significantly in the second part of the study (OR = 1.27, 95% CI = 1.02–1.58, *P* = 0.033). In Kiboga, none of the factors tested was a significant predictor of antimalarial treatment of RDT-positive cases (*P* > 0.2) with 96.7% receiving treatment. A lower proportion of RDT-negative patients received antimalarial treatment in Kiboga District than in the control area. In Kiboga, after introduction of PCWs, antimalarial treatment of RDT-negatives increased for young children but decreased for older patients; whereas in the control district, treatment of negatives increased for all age groups over the same time period.

Records for 39,882 patients seen by community health workers in Uganda were analyzed (Table 7). The odds of conducting an RDT were 1.61 (95% CI = 1.49–1.74) times higher for the post-PCW period compared with the pre-PCW period in both districts. Patients with positive RDT results had twice the odds of receiving antimalarial treatment in Kiboga compared with Kyankwanzi (OR = 2.20, 95% CI = 1.49–3.27), although both districts treated over 99% of RDT-positive cases with antimalarials (Table 7). In Kiboga, the proportion of RDT-negative patients treated with an antimalarial decreased from 35.4% before PCW introduction to 23.3% afterward. In Kyankwanzi, the proportion increased from 20.9% pre-PCW to 60.3% over the same time period.

### Qualitative findings on health workers' perceptions of PCWs.

In Laos, 84 participants (60% community workers) took part in 11 semistructured interviews and 11 FGDs. In Uganda, 119 participants (76% community workers) participated in 29 interviews and 11 FGDs. A more extensive analysis of qualitative data will be reported elsewhere; a summary of key findings is presented herein.

Most health workers reported that difficulties in performing the PCWs were generally minor and became easier with training and experience. Several noted the challenge posed by the appearance of faint—rather than clearly visible—RDT test lines with PCW use (Box 1

Box 1 **Representative quotes from health worker participants in focus group discussions and semistructured interviews**

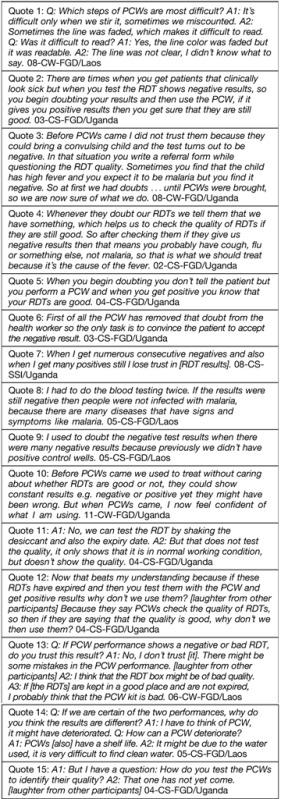

, Quote 1 [Q1]).

In general, PCWs were discussed by health workers as a way to confirm RDT quality and restore confidence in RDT results in some situations where doubts existed. For example, when health workers encountered a discrepancy between their own clinical impression (that a patient had malaria) and a negative RDT result, PCW use was reported to help resolve the uncertainty (Q2 and Q3). Some health workers mentioned their use of PCWs to patients as a way of convincing them that RDT results were reliable (Q4); but more often health workers did not mention PCWs to patients as they believed such information was too technical for patients to understand, or was relevant only for health workers (Q5 and Q6).

In both Laos and Uganda, among both clinic staff and community workers, one of the most frequently mentioned reasons for health workers to doubt RDT results was obtaining “too many” consecutive similar results when testing patients, especially consecutive negative results (Q7). Previously, typical reactions to this concern might have been either to repeat a patient's test to confirm the result (Q8), or to disregard a negative result and treat empirically with antimalarials. PCWs appeared to have some capacity to restore trust for health workers faced with serial negative results (Q9).

Before PCW introduction most health workers recognized that RDTs could be of poor quality or faulty. However, for some, the introduction of PCWs appeared to confirm this possibility (Q10). Similarly, health workers had previously been trained to check RDTs' expiration date and desiccant packet as a means of quality control; whereas PCWs introduced a new quality-control option that needed to be translated into understanding and practice (Q11). However, the availability of multiple quality-control indicators also led some health workers to experiment with expired RDTs (Q12). Finally, some participants questioned whether PCWs could also be of poor quality (Q13–15).

## Discussion

PCWs have been developed as a point-of-care quality-control tool to monitor the validity of malaria RDTs. This study introduced PCWs for use by front-line health workers in Laos and Uganda. In both settings, after a half-day training, most participating clinic staff and community health workers were able to correctly perform PCWs and interpret results, and to maintain these skills over the 6-month study duration. When PCWs were provided at health-care sites for routine use, most participants recorded correct use of PCWs and appropriate actions based on results. There were both quantitative and qualitative evidences in some settings that PCWs improved health workers' confidence in RDT results for patient care.

For PCW use to be effective, users must correctly perform PCW steps and interpret RDTs, and take the appropriate action based on RDT results. PCW steps that appeared most challenging included obtaining and transferring the correct volumes of water and PCW solution, and stirring the solution for the recommended length of time. Similar difficulties with transferring small, precise volumes have been reported in RDT training efforts, especially among lower-level health workers.^11^,^22^,^23^ Significant errors in volume transfer and stirring could lead to too little antigen reaching the RDT, which may result in a “false-negative” result and a false impression that the RDT is defective. Pending any simplification of the PCW format, careful training and supervision may reduce this risk. PCW validation and stability studies are ongoing, and final technical specifications will be reported elsewhere.

Anecdotally, study team observers noted that poor eyesight appeared to contribute to some participants' difficulties preparing PCWs; visual acuity was not assessed systematically, but health worker age (which may be a proxy in some cases) was associated with incorrectly filling the PCW dropper particularly in Uganda. Poor vision may also influence health workers' interpretation of RDT results, especially in the case of faint test lines.^24^,^25^ The amount of antigen in a PCW is intended to differentiate between a valid RDT, and one that cannot detect the lower limits of most clinically significant parasitemia (∼200 parasites/μL)^20^; therefore, PCW solution typically produces a faint RDT test line on a working RDT. Both quantitative and qualitative data indicate that some study participants were uncertain of how to interpret faint test lines, although PCW training had stated that a line of any intensity should be considered positive. Indeed, some health workers intentionally applied more than the recommended volume of PCW solution to achieve a stronger test line.

In general, the few health workers who found one aspect of PCWs challenging (e.g., preparation steps) also made errors with others (e.g., interpretation). Therefore, future PCW implementation programs could plan to identify health workers who may benefit from extra training assistance. The training materials and pictorial guide designed for this study appeared appropriate for the participating front-line health workers. No significant patterns were identified between PCW performance and length of experience with RDTs. Also, no substantial differences between clinic staff and community workers were seen in ability to correctly perform, interpret and use PCWs. In many settings, community health workers (village health volunteers) are tasked with managing malaria with or without RDTs.^26^–^28^ This study provides reassurance that PCWs may also be integrated into such programs.

During the study, all negative or invalid RDT results obtained with PCWs were immediately followed up by telephone with the reporting health worker. Study staff verbally assisted the health worker to repeat the PCW assessment with another RDT from the same stock. In all cases, the repeat test result was positive; there were no confirmed cases of poor-quality RDT stocks identified during the study. In other settings, where poor-quality RDTs may be more common, extra attention may be required to ensure that functional reporting and response systems are in place to handle health workers' reports in a timely way.

Where data are available to assess the effect of PCWs on RDT use and patient management, these appear to be neutral or, in some cases, possibly beneficial. In Laos, antimalarial treatment of RDT-positive patients rose after PCW introduction, but it is unclear whether this effect was due to PCWs or to other factors. In Uganda, after PCW introduction, use of RDTs dropped among clinic staff in both the PCW and control area, whereas it rose among community workers in both areas; no clear explanation (e.g., fluctuations in RDT supply) for these differences was identified. There were no substantial changes in antimalarial treatment of RDT-positives in Uganda. However, after PCW introduction, antimalarial treatment of RDT-negative patients declined significantly for patients older than 5 years managed by clinic staff, and for all patients seen by community workers; this occurred in the face of large increases over the same time period in the control district (and for young children managed by clinic staff in the PCW district). Coupled with qualitative data indicating that PCWs boosted many health workers' confidence in RDT results, these findings suggest that PCWs may help to address the persistent problem of unnecessary antimalarial treatment of test-negative patients.^29^,^30^

At the study end, health workers were asked about their recommendations for future implementation of PCWs (data not shown). Around three-quarters of Lao health workers and two-thirds in Uganda suggested that PCWs should be packaged separate from RDTs to avoid waste and to avoid the risk of exposing both RDTs and PCWs to adverse transport and storage conditions. Health workers who favored packaging PCWs and RDTs together cited convenience as a rationale. Most participants recommended that PCWs should be implemented alongside clear guidelines for when to use them (rather than leaving health workers to design their own schedules).

This study has several limitations. Health workers knew that they were participating in research, so the Hawthorne effect may have influenced their PCW performance under observation as well as records kept during routine work. Keeping written records appeared to be challenging for some study participants, especially in Laos where some records with missing data were excluded from analysis. This observation reflects the challenges of conducting research among front-line health workers in malaria-endemic areas (and also highlights one of the challenges encountered when health-care systems must rely on staff with limited education). More PCWs were used per health worker in Uganda than in Laos, perhaps at least in part because the RDTs in the Uganda study area were more freely available. Patient-level data on RDT use and antimalarial prescribing was only available in Uganda, so the effects seen there could not be compared with data from Laos.

The need for malaria RDT quality-control strategies, appropriate for routine health-care settings in endemic areas, is well recognized.^10^–^13^ Alternatives such as microscopy and molecular tools as reference tests use different biological parameters, do not provide real-time information and are generally not feasible for most programs. Some RDT manufacturers sell positive controls as a separate catalogue item, but these require a cold chain, are product specific, and some are not for single use. Alternatively, researchers have evaluated dried blood containing cultured *Plasmodium falciparum* parasites at specific densities as a positive control for RDTs, but this approach does not generate consistently reproducible antigen concentrations; in addition, the need for cultured parasites, potential for degradation under field conditions, and multiple rehydration steps limit their use.^14^,^16^ If technical specifications are met, including stability under typical storage conditions,^15^ PCWs based on dried recombinant antigen, such as the prototype introduced in this study, appear best suited for wide-scale implementation.

## Conclusions

This is the first study to introduce PCWs for malaria RDTs for routine use by front-line health workers in endemic areas. Over the 6-month study period, health workers were able to correctly prepare and interpret PCW results to identify and report poor-quality RDTs. Results suggest that PCWs may improve health workers' confidence in RDT results, and reduce antimalarial overtreatment of RDT-negative patients. Data collected are intended to guide eventual implementation strategies for PCWs that meet technical specifications. Future work should refine these strategies for various contexts, and evaluate the longer term impact of PCWs on health worker behaviors, patient management, and cost-effectiveness of RDT use. Lessons learned from malaria RDT and PCW implementation may be valuable in introducing other point-of-care diagnostic and quality-control tools.

## Figures and Tables

**Table 1:** Participating health workers: enrolment population and descriptive data

Feature	Lao People's Democratic Republic Number (%) unless otherwise indicated	Uganda Number (%) unless otherwise indicated
No. of participants enrolled	267	290
Age in years: median, interquartile range, range	36, 28–45, 17–73	40, 32–47, 22–69
Female gender	57 (21)	151 (52)
Male gender	210 (79)	139 (48)
Professional category		
Community workers	192 (72)	240 (83)
Clinic staff	75 (28)	50 (17)
Highest educational level achieved*		
Any primary school	118 (45)	125† (43)
Any secondary school	125 (48)	128 (44)
Laos: Diploma/Uganda: Tertiary or University	20 (8)	37 (13)
Formally trained in RDT use	237/265 (89)	277 (96)
If trained, approximate no. of months ago‡: median, interquartile range, range	24, 12–48, 1–120	33, 24–34, 1–60
Has used RDTs in routine patient care	251/264 (95)	288 (99)
If RDTs used, approximate no. of months used§: median, interquartile range, range	36, 15–48, 1–120	32, 24–34, 1–60
Participation—no. of health workers who attended the three study assessments∥		
All: 1, 2, and 3	172 (64)	263 (91)
1 only	26 (10)	10 (3)
1 and 2 only	20 (7)	8 (3)
1 and 3 only	49 (18)	9 (3)

RDT = rapid diagnostic test.

* Data missing for four participants in Laos.

† Includes three who reported no formal education.

‡ Data missing for 56 participants in Laos; for eight in Uganda.

§ Data missing for 50 participants in Laos; for 11 in Uganda.

∥ In Laos, heavy flooding in the study area affected travel conditions and health worker attendance.

**Table 2:** Positive control well performance checklist*

	Lao People's Democratic Republic Number (%)			Uganda Number (%)		
	Study start (*N* = 267)	Midpoint (*N* = 192)	Study end (*N* = 221)	Study start (*N* = 290)	Midpoint (*N* = 271)	Study end (*N* = 272)
Looked at job aid ≥ 3 times while performing PCW	64/266 (24)	68 (35)	63/220 (29)	252/288 (88)	199/270 (74)	144/268 (54)
Looked at job aid 1 and 2 times while performing PCW	55/266 (21)	68 (35)	67/220 (30)	20/288 (7)	58/270 (21)	81/268 (30)
Did not look at job aid while performing PCW	147/266 (55)	56 (29)	90/220 (41)	16/288 (6)	13/270 (5)	43/268 (16)
Six key steps in PCW procedure	Number (%) of health workers performing PCW procedure step correctly					
Fill PCW dropper with water to mark	256 (96)	180 (94)	214/220 (97)	255 (88)	223 (82)	240 (88)
Empty water into PCW tube	262 (98)	183 (95)	218 (99)	286 (99)	262 (97)	252 (93)
Mix solution for 120 seconds	260 (97)	189 (98)	214 (97)	276 (95)	235 (87)	249 (92)
Transfer one drop PCW solution to correct RDT well	257 (96)	176 (92)	216 (98)	282/289 (98)	245 (90)	259 (95)
Put correct no. of buffer drops into correct well	261/266 (98)	187 (97)	217/220 (99)	278 (96)	260 (96)	258 (95)
Wait correct length of time before reading RDT result	264/265 (99.6)	189 (98)	217 (98)	282/289 (98)	267/270 (99)	258/270 (96)
All PCW preparation steps completed correctly	235/264 (89)	158 (82)	204/219 (93)	227/285 (80)	166/266 (62)	188/270 (70)
Read RDT result correctly	248/252 (98)	190 (99)	213/219 (97)	282/287 (98)	264/270 (98)	264/267 (99)
Give a correct/rational explanation for RDT result	253/256 (99)	190/191 (99)	214/219 (98)	281/284 (99)	265 (98)	261/266 (98)

PCW = positive control well; RDT = rapid diagnostic test. Health worker performance of PCW with RDT, observed by study staff, immediately after training at start of study, at study midpoint 3 months after training, and at study end 6 months after training.

* Some observations missing, as indicated by insertion of denominators.

**Table 3:** PCW study participants' interpretation of reacted RDTs, in response to question: “What would you do if you got this result while using a PCW to check the RDT stock at your usual post of work?”*

Study midpoint			Study end		
True result of RDT	Correct proposed action†		True result of RDT	Correct proposed action†	
	Laos (*N* = 188)	Uganda (*N* = 275)		Laos (*N* = 216)	Uganda (*N* = 277)
RDT 1 (positive)	181 (96)	266 (97)	RDT 1 (positive)	210/215 (98)	276 (99.6)
RDT 2 (negative)	185/187 (99)	263 (96)	RDT 2 (positive; faint line)	191/214 (89)	262 (95)
RDT 3 (invalid)	185/87 (99)	266 (97)	RDT 3 (negative)	198/214 (93)	273 (99)
RDT 4 (negative)	183 (97)	264 (96)	RDT 4 (invalid)	208/211 (99)	274 (99)
RDT 5 (positive)	171 (91)	269 (98)	RDT 5 (negative)	199 (92)	273 (99)
Composite: all five responses correct	167/187 (89)	246 (89)	Composite: all five responses correct	166/208 (80)	257 (93)

PCW = positive control wells; RDT = rapid diagnostic test.

* Some observations missing, as indicated by insertion of denominators.

† The correct action in response positive RDT results included continuing to use the stock of RDTs in routine patient care. The correct actions in response to negative or invalid RDT results included repeating the PCW assessment with a second RDT from the same batch, calling the study team or supervisor for advice, or returning the stock of RDTs to a supervisor for replacement.

**Table 4:** Records of positive control well use kept by health workers at their work sites over 6-month study period

Feature	Lao People's Democratic Republic* Number (%)	Uganda Number (%)
No. of health workers who brought PCW use records	221 (83)	275 (95)
Total no. of PCW use records received	762	1685
No. of PCWs used per reporting clinic staff: median, interquartile range, range	3, 2–5, 1–12	7, 5–12, 1–28
No. of PCWs used per reporting community worker: median, interquartile range, range	3, 2–4, 1–7	5, 4–7, 1–20
Recorded reason for performing a PCW (reasons are not exclusive)		
“I received a new stock of RDTs”	481/747 (64)	4,83/1,645 (29)
“I wanted to check the quality of my RDTs”	239 (32)	1,049 (64)
“I have been getting many negative RDT results with patients”	16 (2)	74 (5)
“I'm not sure about the RDT results I am getting”	11 (1)	51 (3)
Other reasons	0	109 (7)
RDT result with PCW		
Positive	711/738 (96)	1,510/1659 (91)
Negative†	24 (3)	142 (9)
Invalid†	3 (0.4)	7 (0.4)
Recorded action in response to PCW result		
Continue using RDT stock with patients	688/723 (95)	1,426/1,651 (86)
Repeat PCW quality check with another RDT	32 (4)	209 (13)
Stop using RDT stock and call supervisor and/or study team	2 (0.3)	31 (2)
“Correct” action based on recorded RDT result	685/709 (97)	1,533/1,626 (94)
“Probably correct” action*	—	77 (5)
“Correct” or “probably correct” action*	—	1610 (99)
“Incorrect” action recorded based on recorded RDT result	24/709 (3)	11 (1)
“Correct” action if RDT recorded as positive	667/683 (98)	1,411/1,488 (95)
“Incorrect” action if RDT recorded as positive	16 (2)	0
“Correct” action if RDT recorded as negative or invalid	18/26 (69)	122/138 (88)
“Incorrect” action if RDT recorded as negative or invalid	8 (31)	11 (8)

PCW = positive control wells; RDT = rapid diagnostic test.

* Many Ugandan participants wrote their action on the record form rather than using the tick boxes. In some cases, this necessitated interpreting the intended action from incomplete phrases, which resulted in categorization as “probably correct” or “probably not correct.”

† All negative or invalid RDT results that were reported to study staff were followed up immediately by telephone. In all cases, when the health worker was verbally assisted to repeat the assessment with the correct procedure using a second RDT from her/his stock, the result was positive. There were no confirmed cases of poor-quality RDT stocks identified during the study.

**Table 5:** EMMs for RDT, results, and antimalarial treatment in Lao People's Democratic Republic health facilities with and without PCWs*

Province	Patient age (years)	EMM for proportion of patients receiving RDT (95% CI*)	EMM for proportion of patients RDT-positive (95% CI*)	EMM for proportion of RDT-positive patients receiving antimalarial treatment (95% CI)
Sekong (control)	0–5	0.331 (0.288–0.380)	0.184 (0.115–0.296)	0.972 (0.955–0.988)
	> 5	0.420 (0.384–0.460)	0.147 (0.119–0.181)	
Salavan (PCW)	0–5	0.397 (0.352–0.447)	0.184 (0.115–0.296)	Pre-PCW: 0.934 (0.922–0.947)
	> 5	0.504 (0.480–0.529)	0.308 (0.286–0.332)	Post-PCW: 0.974 (0.965–0.982)

CI = confidence interval; EMM = estimated marginal mean; PCW = positive control wells; RDT = rapid diagnostic test.

* EMMs are presented individually for groups where significant differences were detected (*P* < 0.05), and are merged across categories when no significant difference between categories was detected.

**Table 6:** EMMs for RDT, results, and antimalarial treatment in Uganda health facilities with and without PCWs*

District	Period	Patient age (years)	EMM for proportion of patients receiving RDT (95% CI*)	EMM for proportion of patients RDT-positive (95% CI*)	EMM for proportion of RDT-positive patients receiving antimalarial treatment (95% CI*)	EMM for proportion of RDT-negative patients receiving antimalarial treatment (95% CI*)
Kyankwanzi (control)	Pre-PCW	< 5	0.858 (0.850–0.865)	0–5 years: 0.403 (0.394–0.412) > 5 years: 0.351 (0.345–0.357)	0.975 (0.971–0.978)	0.302 (0.289–0.316)
		≥ 5	0.830 (0.824–0.836)			0.196 (0.188–0.205)
	Post-PCW	< 5	0.725 (0.715–0.735)		0.980 (0.977–0.983)	0.431 (0.414–0.449)
		≥ 5	0.734 (0.728–0.740)			0.340 (0.331–0.350)
Kiboga (PCW)	Pre-PCW	< 5	0.650 (0.634–0.665)	0.470 (0.458–0.482)	0.967 (0.962–0.971)	0.024 (0.017–0.034)
		≥ 5	0.546 (0.535–0.557)			0.047 (0.039–0.056)
	Post-PCW	< 5	0.545 (0.531–0.559)	0.571 (0.562–0.579)		0.056 (0.044–0.072)
		≥ 5	0.525 (0.518–0.533)			0.019 (0.015–0.023)

CI = confidence interval; EMM = estimated marginal mean; PCW = positive control wells; RDT = rapid diagnostic test.

* EMMs are presented individually for groups where significant differences were detected (*P* < 0.05), and are merged across categories when no significant difference between categories was detected.

**Table 7:** EMMs for RDT, results, and antimalarial treatment in Uganda community work stations with and without PCWs*

District	Period	Patient age (years)	EMM for proportion of patients receiving RDT (95% CI*)	EMM for proportion of patients RDT positive (95% CI*)	EMM for proportion of RDT-positive patients receiving antimalarial treatment (95% CI*)	EMM for proportion of RDT-negative patients receiving antimalarial treatment (95% CI*)
Kyankwanzi (control)	Pre-PCW	< 5	0.869 (0.861–0.877)	0.749 (0.738–0.759)	0.996 (0.995–0.997)	0.209 (0.176–0.246)
		≥ 5		0.616 (0.516–0.707)		
	Post-PCW	< 5	0.915 (0.911–0.919)	0.775 (0.768–0.781)		0.603 (0.564–0.642)
		≥ 5		0.649 (0.552–0.735)		
Kiboga (PCW)	Pre-PCW	< 5	0.843 (0.832–0.853)	0.691 (0.677–0.706)	0.992 (0.989–0.994)	0.354 (0.281–0.434)
		≥ 5		0.546 (0.444–0.645)		
	Post-PCW	< 5	0.896 (0.890–0.902)	0.721 (0.711–0.730)		0.232 (0.201–0.266)
		≥ 5		0.582 (0.480–0.677)		

CI = confidence interval; EMM = estimated marginal mean; PCW = positive control wells; RDT = rapid diagnostic test.

* EMMs are presented individually for groups where significant differences were detected (*P* < 0.05), and are merged across categories when no significant difference between categories was detected.
